# Hamster neogenin, a host-cell protein contained in a respiratory syncytial virus candidate vaccine, induces antibody responses in rabbits but not in clinical trial participants

**DOI:** 10.1080/21645515.2019.1693749

**Published:** 2020-01-17

**Authors:** Ann-Muriel Steff, Chanel Cadieux-Dion, Gaël de Lannoy, Maria Key Prato, Xavier Czeszak, Bruno André, Dominique C Ingels, Marc Louckx, Walthère Dewé, Marta Picciolato, Koen Maleux, Laurence Fissette, Ilse Dieussaert

**Affiliations:** a GSK, Rockville, Maryland, USA; b GSK, Laval, Quebec, Canada; c GSK, Rixensart, Belgium; d GSK, Wavre, Belgium

**Keywords:** Neogenin, respiratory syncytial virus, fusion protein, vaccine candidate, host cell protein, Chinese hamster ovary, RSV-PreF

## Abstract

A recombinant respiratory syncytial virus (RSV) fusion glycoprotein candidate vaccine (RSV-PreF) manufactured in Chinese hamster ovary cells was developed for immunization of pregnant women, to protect newborns against RSV disease through trans-placental antibody transfer. Traces of a host-cell protein, hamster neogenin (haNEO1), were identified in purified RSV-PreF antigen material. Given the high amino-acid sequence homology between haNEO1 and human neogenin (huNEO1), there was a risk that potential vaccine-induced anti-neogenin immunity could affect huNEO1 function in mother or fetus. Anti-huNEO1 IgGs were measured by enzyme-linked immunosorbent assay in sera from rabbits and trial participants (Phase 1 and 2 trials enrolling 128 men and 500 non-pregnant women, respectively; NCT01905215/NCT02360475) collected after immunization with RSV-PreF formulations containing different antigen doses with/without aluminum-hydroxide adjuvant. In rabbits, four injections administered at 14-day intervals induced huNEO1-specific IgG responses in an antigen-dose- and adjuvant-dependent manner, which plateaued in the highest-dose groups after three injections. In humans, no vaccination-induced anti-huNEO1 IgG responses were detected upon a single immunization, as the values in vaccine and control groups fluctuated around pre-vaccination levels up to 90/360 days post-vaccination. A minority of participants had anti-huNEO1 levels ≥ assay cutoff before vaccination, which did not increase post-vaccination. Thus, despite detecting vaccine-induced huNEO1-specific responses in rabbits, we found no evidence that the candidate vaccine had induced anti-huNEO1 immunity in human adults. The antigen purification process was nevertheless optimized, and haNEO1-reduced vaccines were used in a subsequent Phase 2 trial enrolling 400 non-pregnant women (NCT02956837), in which again no vaccine-induced anti-huNEO1 responses were detected.

## Introduction

During their first year of life, 50-70% of infants are infected with respiratory syncytial virus (RSV), and by their second birthday essentially all children have had an RSV infection.^1^ Severe RSV disease occurs most frequently in the age group <6 months of age, in which it is the most common cause of bronchiolitis, frequently leading to hospitalization.^1–5^ Immunization of pregnant women can protect their newborn infants against RSV disease in the early months of life through the transfer of protective maternal antibodies during pregnancy,^6–9^ and maternal vaccination strategies against other diseases (tetanus, pertussis, and influenza) in neonates have demonstrated efficacy and acceptable safety profiles in clinical trials.^10^

RSV fusion (F) surface glycoprotein is responsible for the fusion of cellular and viral membranes, a process mediated by a dramatic conformational change of RSV F from a pre-fusion to a post-fusion state during virus entry. It has been demonstrated that the pre-fusion (PreF) form is the main target of RSV neutralizing antibodies, therefore this conformation is considered the most promising for a protein-based RSV vaccine.^11^ A number of subunit vaccines based on RSV F are currently in development.^12^ As part of a development program aimed at producing a vaccine to protect infants against RSV-associated disease through maternal vaccination in the third trimester, we have developed a candidate vaccine containing recombinant RSV F protein engineered to preferentially maintain prefusion conformation (RSV-PreF).^13^

The RSV-PreF antigen was produced as a secreted and soluble protein in the Chinese hamster ovary (CHO) cell expression system, routinely used for the manufacturing of recombinant protein vaccines. The use of CHO cells offers many advantages, including an efficient production process as well as appropriate protein folding, assembly and post-translational modifications.^14^ However, host-cell proteins (HCPs) are occasionally co-purified with the drug substance.^15–19^ For the RSV-PreF vaccine, we detected trace amounts of a residual HCP, identified as hamster neogenin (haNEO1), which were co-purified with the antigen. Neogenin is a cell surface trans-membrane protein that is ubiquitously expressed in all tissues, where it serves as a multifunctional receptor.^20–22^ The protein has been suggested to play a role in the regulation of diverse developmental processes, including the development of the central nervous system and the regulation of inflammation.^23–25^ HaNEO1 has a close amino-acid sequence homology with the human neogenin (huNEO1) protein (94% amino acid sequence identity; i.e. [no. of identical residues of the alignment/no. of aligned residues] × 100; for EGW02120.1 and NP_002490.2). This suggests that traces of haNEO1 in the final formulated vaccine could be of possible clinical concern if they were to induce antibodies in the human recipient that could cross-react with the endogenous protein. Albeit infrequently, anti-HCP immune responses mounted by human participants have been described for other HCPs.^17,26^ The theoretical risk of inducing anti-huNEO1 antibodies was however considered low, given the high level of homology, the single-dose administration and the tested vaccine formulations (non-adjuvanted or containing a low-potency adjuvant, i.e. aluminum hydroxide [‘alum’]). Nonetheless, the assessment of potential huNEO1-specific immunoglobulin G (IgG) responses was included as a tertiary safety endpoint in a Phase 1 and a Phase 2 trial (studies RSV F-001 and RSV F-020, respectively) evaluating the safety and immunogenicity of a single dose of the RSV-PreF vaccine in healthy men and healthy non-pregnant women, respectively.^27,28^ These trials evaluated non-adjuvanted and alum-adjuvanted formulations containing different RSV-PreF antigen doses.

The objectives of the presented work were to assess whether the haNEO1 HCP had induced IgG antibodies that could cross-react with huNEO1. To achieve this, we developed an enzyme-linked immunosorbent assay (ELISA) to detect anti-huNEO1 IgG responses in serum. We used the assay first to quantify the presence or induction of anti-huNEO1 IgG antibodies in sera from rabbits vaccinated with different RSV-PreF formulations, and then to quantify anti-huNEO1 IgG in serum samples collected from the participants of the clinical trials described above. Finally, we assessed anti-huNEO1 IgG antibody levels in sera collected from non-pregnant women enrolled in a subsequent Phase 2 trial (RSV F-021^29^) in which haNEO1-reduced vaccines were used.

## Materials and methods

### Determination of the haNEO1 concentration in antigen bulk material

The haNEO1 present in the purified antigen bulk was analytically quantified using a qualified LC-MS-based assay. A technical limitation was the lack of a drug substance lot free of haNEO1, which precluded the use of an external calibration curve. The experimental approach comprised therefore the addition of a standard (recombinant huNEO1, lot WMA1140630; AMS-Bio-OriGene, Abingdon, UK) which was added directly to the samples. Spiked solutions were prepared by adding different concentrations of the standard (0.0685%, 0.1370%, 0.2055% and 0.2740% w/w in total protein) to each sample. The spiked samples and one unspiked sample were analyzed in the same analytical run and reported on the corresponding calibration curve. Five neogenin peptides common to both the Chinese hamster (*Cricetulus griseus*) and the standard were quantified to generate (after specific signal extraction using an extracted-ion chromatogram) five calibration curves. The haNEO1 concentration of a sample was equal to the magnitude of the x-intercept in the plot and was calculated by extrapolation of the linear fit. The analytical range of the assay was determined at 0.0685–0.27% (w/w) of haNEO1, with 97-122% accuracy.

### NEO1-specific ELISA

The huNEO1 (OriGene Technologies, Inc. Rockville, MD, USA) produced in *E. coli*, was adsorbed at 1 µg/mL in phosphate-buffered saline (PBS), overnight at 4°C onto 96-well flat-bottom microtiter plates (50 µL/well). The plates were rinsed three times in wash buffer (0.05% Tween-20/0.9% NaCl), then saturated by incubating for 1 h at room temperature with 100 µl/well diluent buffer (1% bovine serum albumin, 0.1% Tween-20, 0.2% ProClin 300 [Merck KGaA, Darmstadt, Germany] in PBS) and further rinsed three times in wash buffer. Human serum samples were tested in serial 2-fold dilutions (50 µL/well) starting at 1/20 in the diluent buffer containing 5% negative serum. The standard curve was prepared using concentrations ranging from 200–1.56 ng/mL of the standard (affinity purified rabbit anti-huNEO1 polyclonal antibody; 20246-1-AP; Proteintech Group, Inc. Rosemont, IL, USA). Samples, standards and controls were incubated with agitation for 1 h at room temperature. The same procedure was used for rabbit serum samples. After rinsing steps as above, plates were incubated with agitation for 1 h at room temperature with the following secondary antibodies in diluent buffer: for rabbit samples, HRP-conjugated goat anti-rabbit IgG (Merck KGaA, Darmstadt, Germany) and for human samples, HRP-conjugated mouse anti-human IgG cross-reactive with rabbit IgG (05–4220, Invitrogen Waltham, MA, USA). Then, after rinsing steps as above, the plates were incubated for 30 min at room temperature in the dark with prewarmed tetramethylbenzidine substrate (Bio-Rad Hercules, CA, USA). The peroxidase reaction was stopped with 0.5M H_2_SO_4_. The IgG concentration was measured at 450 nm wavelength and calculated by comparison with the standard curve of each plate using Softmax Pro four-parameter curve-fit software (Molecular Devices, LLC, Sunnyvale, CA, USA). The lower limit of quantification (LLOQs) were established at 100 ng/mL and 55 ng/mL for rabbit and human serum samples, respectively.

### Rabbit study

##### Ethical statement

All procedures were conducted at Institut Armand Frappier (Laval, Québec, Canada) according to the guidelines of the Canadian Council on Animal Care and the GSK Policy on the Care, Welfare and Treatment of Animals. Prior to initiation of the study, the protocol was submitted for ethical review and approved by the Institutional Animal Care Committee of the Institut Armand Frappier (approval no. LVL/1205-04/01/A).

##### Husbandry

Female New-Zealand White rabbits (N = 42) were housed individually in cages with perforated floors in a dedicated air-conditioned facility. They were provided *ad libitum* a standard rabbit diet and domestic-quality drinking water.

##### Study design

The rabbits were randomly allocated to 7 treatment arms (N = 6/arm) representing one control (PBS) and 6 different formulations of the RSV-PreF vaccine, either adjuvanted with alum (500 µg/dose) or non-adjuvanted (Table 1). The RSV-PreF antigen was derived from either Good Manufacturing Practices clinical bulk lots or non-clinical pharmacology toxicology bulk lots. All test items were obtained from GSK, Rixensart, Belgium. Each rabbit received 4 intramuscular 0.5-mL injections administered two weeks apart (Days 0, 14, 28 and 42). Serum samples were prepared from blood collected on each treatment day before vaccine administration and on Days 56 (14 days post-dose 4) and 70 (28 days post dose 4). At each blood-sampling time-point, anti-huNEO1 antibody concentrations in serum were assessed by ELISA, as described above.

**Table 1.** Study designs.

			Treatments					
Study (name)	Subjects	Treatment day (all groups)	Group	N	µg RSV-PreF per 0.5 mL dose	Adju-vant	RSV PreF DS lot*	Serology (Day; all groups)
Nonclin. pharma- cology	Rabbits (N = 42)	0, 14, 28, 42	10-Plain-C	6	10	-	CLIN1	0, 14, 28, 42, 56, 70
			10-Alum-C	6	10	Alum	CLIN1	
			60-Plain-C	6	60	-	CLIN1	
			60-Alum-C	6	60	Alum	CLIN1	
			60-Plain-T	6	60	-	TOX	
			60-Alum-T	6	60	Alum	TOX	
			Placebo	6	0	-	-	
Phase 1 (RSV F-001)	Healthy men (N = 128)	0	10-Plain	16	10	-	CLIN1	0, 7, 30, 60, 180, 360
			10-Alum	16	10	Alum	CLIN1	
			30-Plain	15	30	-	CLIN1	
			30-Alum	16	30	Alum	CLIN1	
			60-Plain	16	60	-	CLIN1	
			60-Alum	16	60	Alum	CLIN1	
			Control 1/2	33	0	-	-	
Phase 2 (RSV F-020)	Healthy non-pregnant women (N = 500)	0	30-Plain	126	30	-	CLIN1	0, 30, 60, 90
			60-Plain	124	60	-	CLIN1	
			60-Alum	125	60	Alum	CLIN1	
			Tdap	125	0	-	-	
Phase 2 (RSV F-021)	Healthy non-pregnant women (N = 400)	0	30-Plain	100	30	-	CLIN2	0, 30
			60-Plain	99	60	-	CLIN2	
			120-Plain	99	120	-	CLIN2	
			Placebo	102	0	-	-	

Alum, aluminum hydroxide (500 μg). *Names of the formulated drug substance (DS) lots used in the clinical (‘CLIN’) and nonclinical pharmacotoxicology (‘TOX’) studies are presented. CLIN1 and CLIN2 lots were formulated with different ha-NEO content (lower in CLIN2), and a different DS lot was used for TOX. Placebo control was phosphate-buffered saline. Control 1/2 groups (N = 17/16) received saccharose-NaCl solution. Tdap, vaccine against tetanus, diphtheria, and acellular pertussis (Boostrix, GSK), adjuvanted with 300 µg (US sites) or 500 µg (non-US sites) aluminum hydroxide. N, number of participants per group.

##### Statistics

Descriptive statistical analyses were performed using SAS version 9.1 (SAS Institute Inc., NC, USA). Values below the LLOQ were assigned the value of the LLOQ (100 ng/mL).

### Human serum samples

#### Study designs

Human serum samples were obtained from three previously conducted randomized, placebo-controlled and observer-blind clinical studies evaluating different formulations of the RSV-PreF vaccine.^27–29^ The studies included a Phase 1 multicenter clinical trial evaluating 128 healthy 18–45 years old male participants living in Canada (NCT01905215/RSV F-001), and two multicenter, Phase 2 clinical trials evaluating in total 900 healthy non-pregnant 18–45 years old women (i.e., one trial enrolling 500 women living in Australia, Czech Republic, Germany or the USA [NCT02360475/RSV F-020], and a second trial enrolling 400 women living in Belgium, Estonia, France or Germany [NCT02956837/RSV F-021]). The three studies were approved by the respective institutional Ethics Committees and conducted in accordance with Good Clinical Practice guidelines, the Declaration of Helsinki and applicable regulatory requirements. Written-informed consent was obtained from each participant prior to the collection of all blood samples used in the studies. All treatment arms in the three studies were well balanced in terms of demography. Seven, 30, and 8 participants in the RSV F-001, RSV F-020 and RSV F-021 trials, respectively, withdrew before study completion, but none of the withdrawals was due to an adverse event.

The Phase 1 trial was a first-time-in-human dose-escalation study which evaluated six non-adjuvanted or alum-adjuvanted vaccine formulations containing 10, 30 or 60 µg RSV-PreF (the 10-Plain, 10-Alum, 30-Plain, 30-Alum, 60-Plain, 60-Alum groups), or placebo control (the Control 1 and 2 groups; Table 1). Staggered dose-escalation was performed: participants of the 10-Plain, 10-Alum, 30-Plain, 30-Alum and Control 1 groups were vaccinated first, and participants of the 60-Plain, 60-Alum, and Control 2 groups were vaccinated in a second step. Formulations evaluated in the RSV F-020 trial included the active control vaccine Tdap (reduced-antigen-content diphtheria-tetanus-acellular pertussis; *Boostrix;* GSK) and three RSV-PreF formulations (the 30-Plain, 60-Plain, and 60-Alum groups), while the RSV F-021 trial evaluated three non-adjuvanted RSV-Pre-F formulations (30-Plain, 60-Plain, 120-Plain) and placebo control. In the three trials, all vaccines and controls were administered as a single intramuscular 0.5-mL dose on Day 0.

All vaccine formulations investigated in the three trials were well tolerated. In RSV F-001, fatigue and mild transient injection site pain were the most reported symptoms in all study groups. Fever was uncommon and of low grade. Reported safety and reactogenicity were similar between the vaccine formulations, and no vaccine-related severe adverse events (AEs) or withdrawals occurred. In study RSV F-020, the nonadjuvanted RSV-PreF candidate vaccines were less reactogenic (especially in terms of injection site pain) than Tdap, which was used as a control for local and systemic AEs (of note, Tdap vaccination is considered the standard of care during pregnancy in many countries). Exploratory analyses showed a trend for higher reactogenicity in the 60-Alum group as compared with the nonadjuvanted groups. In study RSV F-021, safety and reactogenicity profile were comparable between RSV-PreF vaccine groups. Injection site pain was more frequently reported by participants receiving RSV-PreF formulations than by those receiving the control vaccine. No vaccine-related SAEs were reported in any study group. Altogether, no safety concerns that would preclude continued or future development of the RSV-PreF vaccine candidate, were identified.^28^

#### Statistics

Since the anti-huNEO1 humoral immune response was considered a safety parameter, evaluations for the current research work were performed on the participants of the Total Vaccinated Cohorts of the three studies for whom immunological results were available. Descriptive statistics of the huNEO1-specific antibody responses were computed using SAS version 9.2 (SAS Institute Inc., NC, USA). Seropositivity was defined as an IgG concentration ≥ 55 ng/mL (LLOQ). Concentrations below the LLOQ were assigned the value 27.5 ng/mL for calculation purposes.

## Results

### Quantification of haNEO1 in the antigen bulk materials

The presence of trace amounts of haNEO1 in the purified RSV-PreF antigen drug substance was initially detected by Western blot assay, and then quantified using a liquid chromatography–mass spectrometry (LC-MS)-based assay. The analyses performed on an antigen drug substance lot that was used to formulate two vaccines evaluated in the preclinical toxicology studies (TOX1 and TOX2; Table 1) revealed a haNEO1 content of 0.78% w/w (7833 ppm, or 470 ng per 60 µg RSV-PreF dose). This amount was 1.8-fold higher than the haNEO1 amounts measured in the drug substance used in the Phase 1 and 2 (RSV F-001 and RSV F-020) studies (i.e., 0.43% w/w [4333 ppm], or approximately 260 ng haNEO1 per 60 µg RSV-PreF).

### Anti-huNEO1 IgG ELISA development and performance

As a first step assay development was undertaken, since no suitable assay quantifying anti-huNEO1 IgG levels in human serum was available. Initial attempts to develop an ELISA using the typical anti-drug antibody format^30^ proved unsuccessful. A number of the human serum samples exhibited high optical density signals, complicating the establishment of a cutoff. Moreover, binding inhibition experiments performed with recombinant huNEO1 suggested the presence of nonspecific binding or cross-reactivity. We therefore developed an indirect ELISA for this purpose. Since no international reference preparation was available for the quantification of anti-huNEO1 IgG in serum, commercially available affinity-purified anti-huNEO1 antibodies derived from the rabbit and goat were tested as potential standards in the assay. The secondary antibody was selected on the premise that it needed to detect both rabbit and human IgG, or both goat and human IgG, in a similar manner. Considering the additional intended use of the ELISA in experiments with rabbit serum as well as the similar performance of the goat and rabbit anti-huNEO1 antibodies, the rabbit anti-huNEO1 antibody was selected as standard. The secondary antibody, purified monoclonal mouse anti-human IgG conjugated to horse-radish peroxidase (HRP), was shown to cross-react with rabbit IgG and recognize human IgG and rabbit IgG with comparable sensitivity (Figure S1).

The (intra-assay) repeatability and (inter-assay) intermediate precision were determined using a human serum pool spiked with affinity-purified rabbit anti-huNEO1 antibodies. Measurements taken over three days and by two operators revealed comparable overall precision for huNEO1 concentration determination in 8-fold or 4-fold serial dilutions (coefficients of variation [CVs]: 22.44% or 22.65%, respectively), both meeting the intermediate precision criterion of CV ≤ 25%. At the lowest rabbit anti-neogenin IgG concentration tested (110 ng/mL), the CV only slightly exceeded this criterion (26.5%). For this reason, and to ensure maximal assay sensitivity, the LLOQ was established at half this concentration, i.e., 55 ng/mL. For calculations, human samples with anti-huNEO1 levels < LLOQ were assigned a value of 27.5 ng/mL. The upper limit of quantification was established at 2500 ng/mL. Specificity tests using huNEO1 and the negative control (an unrelated recombinant protein produced in *E. coli*) revealed 91.9% and 9.5% inhibition of antibody binding, respectively, meeting their respective acceptance targets of ≥ 80% and ≤ 20% (data not shown). Consequentially, the assay was considered suitable for its intended purpose.

### Adjuvant and antigen-dose-dependent anti-huNEO1 antibody responses in rabbits

We first evaluated the immunogenicity of haNEO1 in different formulations of the RSV-PreF vaccine in the rabbit model. The formulations included antigen derived from either clinical (‘C’) or non-clinical toxicology (‘T’) drug substance lots (see Table 1 for group acronyms), with or without alum. Rabbits received RSV-PreF vaccine or PBS control four times every two weeks, and anti-huNEO1 antibody concentrations were determined at different time points before and after vaccination, as detailed in Table 1.

**Figure 1.**
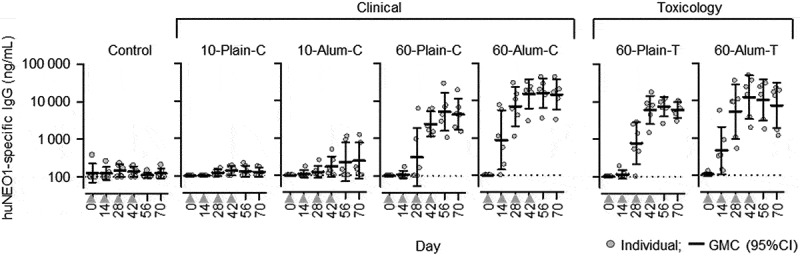
Anti-neogenin antibody concentrations in rabbits.

At baseline, quantifiable anti-huNEO1 IgG levels (≥100 ng/mL for this model) were detected in three of the 42 rabbits (7%; one in the control group, at 380 ng/mL, and two in the group receiving alum-adjuvanted 60 µg RSV-PreF from the toxicology lot [60-Alum-T group], at 110 and 130 ng/mL; Figure 1). In the control group, four rabbits had quantifiable anti-NEO1 IgG concentrations over the course of the study. Among the groups receiving antigen from the clinical lots, only the 60-Alum-C group exhibited increased levels two weeks after the first dose (Day 14), which further increased two weeks after the second immunization (Day 28). At this time-point, most animals of the 60 µg dose groups had anti-huNEO1 IgG levels above the cutoff value. Geometric mean concentrations (GMCs) in these groups generally peaked at Day 42 (two weeks post-third immunization) and plateaued at later time-points. In contrast, for the recipients of 10 µg RSV-PreF (with or without alum) only a few animals exhibited (slightly) increased levels at Days 14 and 28, and GMCs remained overall low throughout the study (though slight increases were observed in the 10-Alum-C group two and four weeks post-last dose [Days 56 and 70]). A similar dose-response was observed for groups receiving antigen from the toxicology lots (60-Plain-T and 60-Alum-T groups), in which the levels reached a plateau at Day 42.

Collectively, the results suggest that the trace amount of haNEO1 in the toxicology and clinical RSV-PreF drug substance lots was capable of inducing IgGs that recognized huNEO1 antigen in rabbits.

### No evidence of vaccination-induced anti-huNEO1 immunity in healthy adults

The same ELISA was then used to evaluate whether the haNEO1 traces contained in the vaccine candidate had induced huNEO1-specific IgG responses in participants of a Phase 1 trial enrolling 128 adult men (15–17 per study group; study RSV F-001^27^) and of a Phase 2 trial enrolling 500 non-pregnant women (124–126 per study group; study RSV F-020^28^). Both trials had a randomized, controlled, observer-blind design and evaluated the safety and immunogenicity of different RSV-PreF formulations (see Table 1 for study designs and group acronyms).

#### Phase 1 trial in healthy men (RSV F-001; NCT01905215)

**Figure 2.**
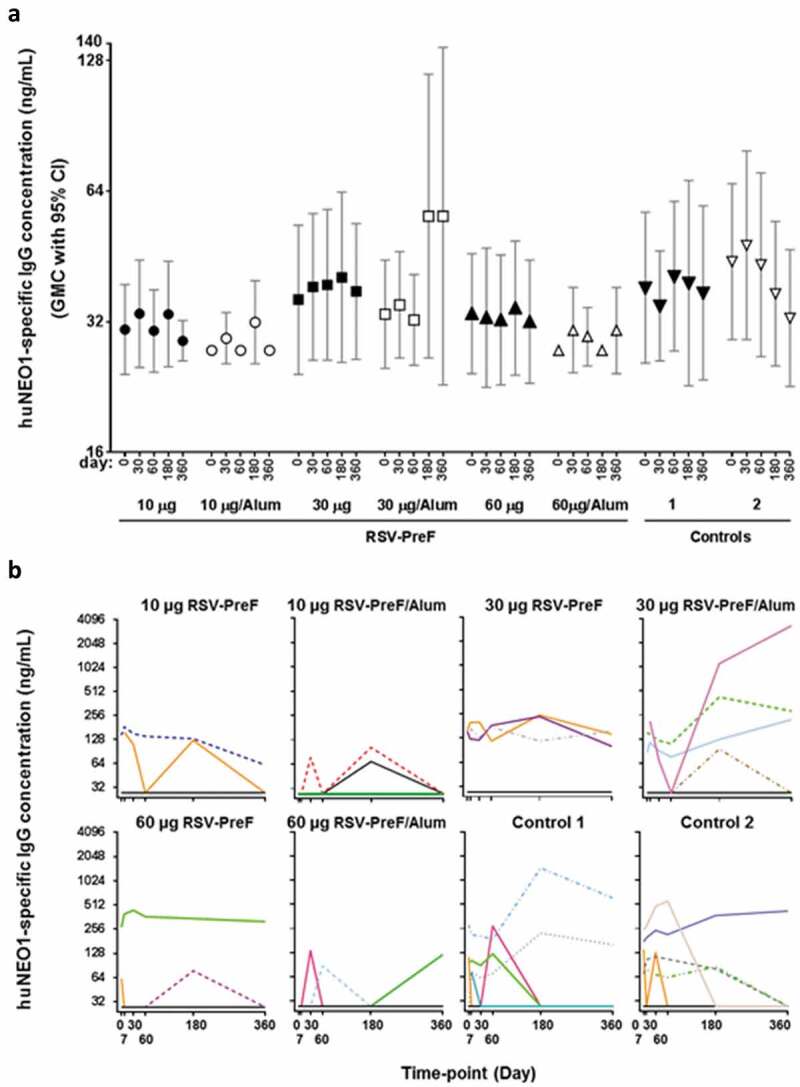
Anti-neogenin antibody concentrations in healthy men.

Before vaccination, 15 participants (12%) had quantifiable anti-huNEO1 IgG concentrations, ranging from 63 to 284 ng/mL. After vaccination, no clear differences between pre- and post-vaccination GMCs were seen in any of the investigational and control groups (Figure 2(a)). Based on the overlapping 95% confidence intervals (CIs), there was neither any evidence for differences in the distribution of GMCs between the vaccinated and control groups, nor an indication of an antigen-dose or adjuvant effect. In a minority of participants across all groups, individual anti-huNEO1 levels fluctuated around pre-vaccination levels over time (Figure 2(b)). In some participants, the anti-huNEO1 concentration after vaccination was higher compared to the pre-vaccination level on at least one post-vaccination time-point, but this was observed irrespective of the treatment group (i.e., in 7/31 [22.6%] and 13/91 [14.3%] of participants in the control and RSV-PreF groups, respectively). One individual in the 30-Alum group exhibited particularly increased levels at Days 180 and 360, after showing undetectable levels of antibodies on Day 60. Because a similar pattern was also observed in both control groups, it is considered unlikely that these late increases were induced by vaccination. There was therefore no evidence to suggest that any of the RSV-PreF formulations had induced a huNEO1-specific IgG response in these participants.

#### First phase 2 trial in healthy non-pregnant women (RSV F-020; NCT02360475)

**Figure 3.**
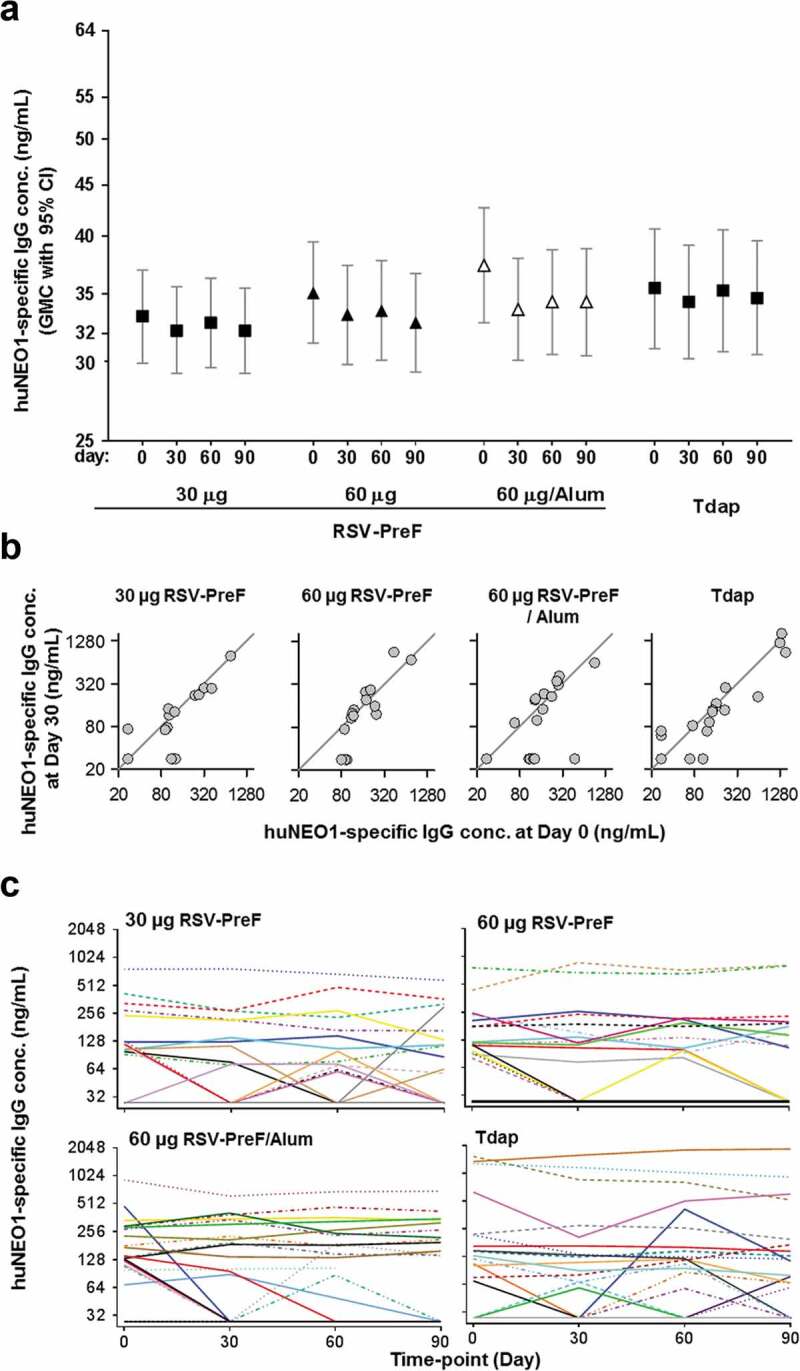
Anti-neogenin antibody concentrations in healthy non-pregnant women.

Anti-huNEO1 IgG concentrations were also measured in samples from a multicenter Phase 2 trial enrolling 500 healthy non-pregnant women who received one dose of either an RSV-PreF vaccine (30-Plain, 60-Plain or 60-Alum), or control (tetanus, diphtheria and acellular pertussis [Tdap]) vaccine at Day 0.^28^ Results were overall similar to those observed in the Phase 1 trial. Sixty-five participants (13%) had quantifiable anti-huNEO1 antibody levels prior to vaccination, ranging from 68 to 1555 ng/mL. Moreover, there were no significant differences between the Day 0 and post-vaccination (Days 30, 60 or 90) values across all groups, nor was there any evidence of an antigen-dose or adjuvant effect (Figure 3(a)). Several participants were seropositive (anti-huNEO1 IgG levels ≥ 55 ng/mL) on at least one time-point before or after vaccination (N = 19, 17, 21 and 22 in the 30-Plain, 60-Plain and 60-Alum and Tdap groups, respectively; Figure 3(b)). As in the Phase 1 trial, levels of anti-huNEO1 antibodies fluctuated in some participants during the study. There were only three cases (of which two in the control group) in which levels had increased by more than two-fold at Day 30, all of which reflected a transition from below to above the LLOQ (but all remaining below 73 ng/mL). For most of the seropositive participants and irrespective of the treatment group, the individual levels after vaccination remained similar to those at pre-vaccination (Figure 3(c)).

**Figure 4.**
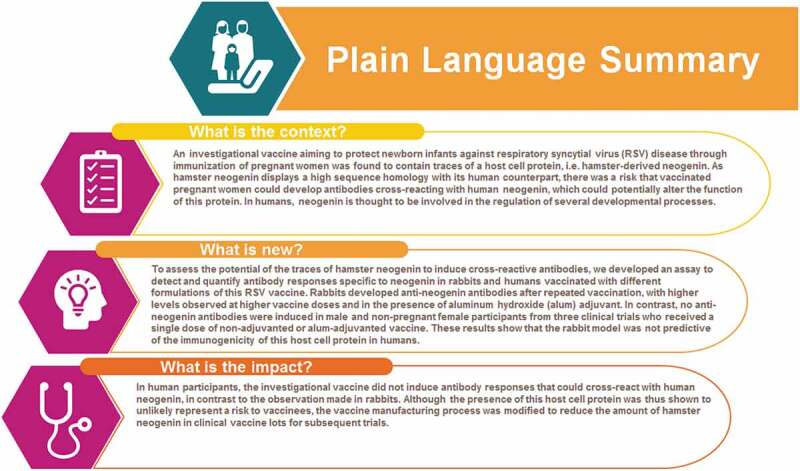
Plain language summary.

Overall, the data from both clinical trials indicated that in most participants, anti-huNEO1 IgG levels were below the quantification limit at all time points including at pre-vaccination, while in a minority of participants, these levels fluctuated over time, irrespective of the treatment group.

#### Second phase 2 trial in healthy non-pregnant women (RSV F-021; NCT02956837)

Despite the lack of evidence of vaccine-induced anti-huNEO1 IgG responses in the two preceding clinical trials, the antigen purification process was modified to further reduce the haNEO1 levels. Following these modifications, the levels of residual haNEO1 in clinical lots were below the LLOQ of the LC-MS-based assay (i.e., < 0.0685% w/w [< 685 ppm]; or < 0.08 µg haNEO1 per 120 µg RSV-PreF). The haNEO1-reduced drug substance material was used to produce the vaccine formulations evaluated in a second multicenter Phase 2 trial.^29^ This trial (RSV F-021) enrolled 400 healthy non-pregnant women who received non-adjuvanted formulations containing one of three different antigen doses (30, 60 or 120 µg RSV-PreF), or PBS (Table 1).

Consistent with the previous observations, anti-huNEO1 IgG responses (range: 56 to 848 ng/mL) were detected at pre-vaccination in a minority of participants (N = 37; 9%), and no evidence for a vaccine-induced response was observed (Figure S2).

## Discussion

Trace amounts of process-derived haNEO1 protein were identified in the purified drug substance of an RSV-PreF maternal vaccine candidate. Given the 94% amino sequence identity between haNEO1 and huNEO1, and the suggested role of neogenin in central nervous system development amongst other functions,^23–25^ a putative vaccine-induced anti-huNEO1 IgG response could have posed a safety risk upon RSV-PreF vaccine administration during pregnancy. In preparation for clinical trials in pregnant women, we investigated whether the RSV-PreF formulations derived from this drug substance material induced anti-huNEO1 antibody responses, first in an animal model and then in humans (using serum samples from two clinical RSV-PreF vaccine trials enrolling healthy men and healthy non-pregnant women^27,28^). We found that, although haNEO1 appeared capable of inducing IgG antibodies recognizing huNEO1 in rabbits, there was no evidence of a link between vaccination and increased huNEO1-specific antibody responses in the clinical trial participants. The progression from experimental studies in rabbits, to clinical studies conducted first in men, and then in non-pregnant women of child-bearing age, was a cautious approach to detect potential safety issues linked to the induction of an immune response to huNEO1, before moving to the target population for this vaccine, i.e., pregnant women. Our data clearly indicate that the observations made in rabbits were not predictive of the human response, since no anti-huNEO1 response could be detected in the three clinical trials conducted. The risk of inducing a potentially detrimental anti-huNEO1 response in pregnant women was therefore considered minimal. As an additional safety precaution, two developmental toxicity studies of the RSV PreF vaccine formulations (with or without alum) were conducted early during the vaccine development, one in rats and one in rabbits, under Good Laboratory Practices conditions. Under the defined experimental conditions and in both animal species, the RSV-PreF candidate vaccine formulations did not adversely affect female fertility, embryo-fetal (including teratogenicity) development, pre- and post-natal survival and early postnatal development (unpublished data).

In rabbits, the magnitude of vaccine-induced anti-huNEO1 responses was a function of (i) the number of injections administered, with a plateau being reached after three doses; (ii) the RSV-PreF antigen dosage, with a higher induction of antibodies in animals receiving the highest (60 µg) RSV-PreF dose; and (iii), the presence of alum adjuvant in the vaccine formulation, with higher and earlier responses observed in the adjuvanted groups. In humans however, neither a higher antigen dose nor the presence of adjuvant triggered the generation of anti-huNEO1 antibodies upon a single vaccine administration. The fact that we were able to detect anti-huNEO1 IgG in rabbits, in a dose- and adjuvant-dependent manner, with the exact same assay as the one that was used to detect such responses in human sera, suggested that the absence of detectable levels in humans was not linked to a technical issue. In addition, it is noted that this apparent discrepancy between animal and human immunogenicity data is not unusual,^31,32^ since animals do not always recapitulate human immune responses. Besides the general differences observed in immune responses observed between humans and animals (in this case rabbits), we hypothesize that more specific mechanisms linked to the vaccine under consideration may, at least partially, explain this result. Although both humans and rabbits are in principle naïve to haNEO1, the two species differ in their serological status for RSV, since all adult humans, but not rabbits, have been exposed to RSV multiple times.^1,33^ Upon vaccination of RSV-primed humans, the abundant preexisting RSV F-specific memory B cells might have outcompeted the naïve huNEO1-specific B cells. In the rabbits however, this would not be the case because they would be naïve for both proteins, thus eliminating the competitive advantage of RSV-specific memory B cells. This could have resulted in different NEO1-specific antibody response levels between the two species. Such initial difference could then have been further amplified by the vaccine dosages, given that the rabbits received four full human doses, each representing (after correction for body weight) a 14-fold higher dose than that used in humans. Furthermore, tolerance-induction mechanisms may be different between both species, although it remains unclear whether the induction of NEO1-specific antibodies in the rabbit would have been dependent on breaking immune tolerance to rabbit neogenin. Of note, the rabbit neogenin isoform (which shares 89% amino acid sequence identity with both huNEO1 and haNEO1) was not used in the ELISA. Additional research, including competition experiments with neogenin from the different species, in depth analyses of B cell responses or use of human-immune-system mice,^34^ including mice with or without preexisting RSV immune responses, may help clarifying the underlying mechanisms.

The results from the three clinical studies showed that some individuals had preexisting anti-huNEO1 antibody responses before vaccination. In some of the participants, anti-huNEO1 antibodies levels fluctuated after vaccination, but the same pattern was also observed at various time-points in participants from the control groups. Two possible, not mutually exclusive explanations are proposed. First, the ELISA may have permitted the detection of IgGs generated by one or more unknown antigen(s) with a weak cross-reactivity with huNEO1. Although the specific inhibition experiments performed in the context of the assay validation suggested that the ELISA could detect anti-huNEO1 IgGs with 92% specificity, this also implies that a low proportion of antibodies could not be outcompeted by the huNEO1 protein. A second explanation may be that the responses reflected naturally present huNEO1-specific antibodies. Though not frequently reported and not described for huNEO1 specifically, naturally present serum IgG responses to human host proteins were detected previously in the context of a peptide microarray vaccine study.^35^ In that study, such responses in healthy individuals were mapped at various time-points including at pre-vaccination. The fact that we have not been able to investigate the underlying cause of the preexisting responses observed in the RSV-PreF vaccine trials is a limitation of the current research work.

Both clinical trials that evaluated the formulations derived from the initial antigen material (studies RSV F-001 and RSV F-020) clearly demonstrated that anti-huNEO1 antibodies were not induced by the RSV-PreF vaccine, even in the presence of adjuvant and at the highest antigen doses. The similarity of results in both trial populations, combined with the large sample size including 128 men and 500 non-pregnant women of child-bearing age, suggest that this finding might be generalizable to a larger population. Despite the lack of haNEO1 immunogenicity observed in the first two trials, we implemented purification steps to further reduce the haNEO1 levels in the drug substance. A third clinical trial (RSV F-021), using haNEO1-reduced lots but also a higher vaccine dosage, further confirmed these results.

The ICH guideline Q3A indicates that impurities in drug substances should be identified at levels exceeding 0.10% or 1000 ppm,^36^ and the amount of haNEO1 in the drug substance used for the first clinical vaccine lots (4333 ppm) exceeded this level. While 100 ppm (0.01%) is the level of HCP typically considered acceptable for a biotherapeutic product, this threshold is generally used for monoclonal antibodies.^15,19^ No safety limits for HCP levels have been defined by regulatory authorities, and the European Medicines Agency specifies the appropriate HCP levels on a case-by-case basis.^37^ We therefore assessed the safety profile of the vaccines formulated from this drug substance in preclinical and clinical studies, while further developing the purification process to reduce haNEO1 levels, accomplishing levels below 0.068% in the subsequently produced lots.

The work presented herein is one of the few occasions where the immunogenicity of an HCP has been directly measured in a large sample size of clinical trial participants. Our data show that for all the RSV-PreF vaccine formulations tested, the haNEO1 HCP did not induce huNEO1-specific IgG responses in healthy non-pregnant adults. Nonetheless, our study was limited by the fact that the use of huNEO1 antigen in our ELISA precluded the detection of any IgGs that were directed to haNEO1 and did not cross-react with the human isoform. However, it seems reasonable to assume that antibodies specifically recognizing haNEO1 would unlikely affect the biological function of huNEO1. Another study limitation is that antibody isotypes other than IgG (such as IgM or IgE) were not measured. We focused on IgGs because, among the five isotypes of human antibodies, only IgG is transferred in significant quantities across the placenta,^38^ where it could potentially affect fetal neogenin function. Altogether, given the presented immunogenicity data and the implemented modified vaccine purification processes, the potential risks associated with the presence of the neogenin HCP were considered negligible. The data therefore support the potential development of a non-adjuvanted RSV-PreF vaccine for use in pregnant women. A plain language summary of the work presented here is provided in Figure 4.

## Supplementary Material

Supplemental Material

## Supplementary Material

The supplemental data for this article can be accessed online at https://doi.org/10.1080/21645515.2019.1693749.
