# Supplementary material for: Hamster neogenin, a host-cell protein contained in a respiratory syncytial virus candidate vaccine, induces antibody responses in rabbits but not in clinical trial participants
Source: Hum Vaccin Immunother. 2020 Jan 17;16(6):1327–37. doi: 10.1080/21645515.2019.1693749 (PMC7482880; doi:10.1080/21645515.2019.1693749)
Supplement: Supplemental Material [file KHVI_A_1693749_SM0620.docx]

# Supplementary Figure

# Figure S1. Human versus rabbit IgG recognition by the secondary antibody (mouse anti-human IgG) used in the anti-human neogenin IgG assay

Commercial human and rabbit immunoglobulins G (IgGs) were plated at concentrations ranging from 0.125 to 8 µg/mL overnight at 4°C onto 96-well flat-bottom microtiter plates (50 µL/well). The plates were rinsed, saturated by incubating for 1 h at room temperature with 100 µl/well diluent buffer (1% bovine serum albumin, 0.1% Tween-20, and 0.2% ProClin 300 in phosphate-buffered saline), and further washed. Plates were then incubated with agitation for 1 h at room temperature with horseradish peroxidase-conjugated mouse anti-human IgG cross-reactive with rabbit IgG (05-4220, Invitrogen Waltham, MA, USA). After washing, the plates were incubated for 30 min at room temperature in the dark with prewarmed tetramethylbenzidine substrate (Bio-Rad Hercules, CA, USA). The peroxidase reaction was stopped with 0.5M H_2_SO_4_. Optical density (OD) values at 450 nm for the binding to human or rabbit IgG were plotted against the IgG concentrations. The graph below shows that there was similar recognition of IgG from both species.


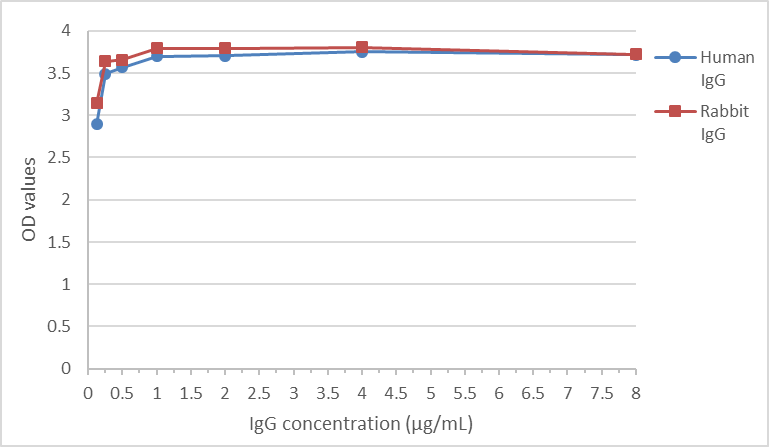


# Figure S2. Anti-neogenin antibody concentrations in healthy non-pregnant women who received RSV-PreF vaccine derived from hamster neogenin-reduced antigen lots

Analyses were performed on the Total Vaccinated Cohort (TVC) of a Phase 2 trial conducted in 400 healthy non-pregnant women[^27^](#_ENREF_27) (NCT02956837). Participants received a single vaccine dose of either a non-adjuvanted RSV-PreF vaccine (30, 60 or 120 µg RSV-PreF) derived from hamster neogenin-reduced drug substance material, or phosphate-buffered saline control on Day 0. (**A**) Geometric mean concentrations (GMCs) with 95% confidence intervals (CI) of human neogenin (huNEO1)-specific IgG responses were measured at pre-vaccination (Day 0) and Day 30 after vaccination. Seronegative samples were assigned a value of 27.5 ng/mL (half the lower limit of quantitation) for calculations. (**B)** Anti-huNEO1 antibody concentrations up to Day 30 are shown by subject. Each line represents an individual subject of the TVC. A total of 358 participants (90%) were seronegative at all time-points, as represented by the horizontal bottom lines in the graphs.
